# Acute Gamma-Hydroxybutyrate (GHB) Intoxication Requiring Hemodialysis in Southern Brazil: A Case Report

**DOI:** 10.7759/cureus.87728

**Published:** 2025-07-11

**Authors:** Mateus Rodrigues Alessi, Gabriel Felipe Contin de Oliveira, Gabriella Shinmi, Matheus L Castro, Valentina G Ramos, Guilherme Triches, Fabiola Bremer, Alan Homero dos Santos, Sivan Mauer

**Affiliations:** 1 Department of Medicine, Universidade Positivo, Curitiba, BRA; 2 Department of Medicine, Hospital Universitário Evangélico Mackenzie, Curitiba, BRA; 3 Department of Medicine, Faculdade Evangélica Mackenzie do Paraná, Curitiba, BRA; 4 Department of Internal Medicine, Hospital Universitário Evangélico Mackenzie, Curitiba, BRA; 5 Department of Nephrology, Hospital Universitário Evangélico Mackenzie, Curitiba, BRA; 6 Department of Psychiatry, Faculdade Evangélica Mackenzie do Paraná, Curitiba, BRA

**Keywords:** alcohol use, ghb intoxication, illicit drugs, respiratory depression, substance recreational use

## Abstract

Gamma-hydroxybutyrate (GHB) is a central nervous system (CNS) depressant with psychoactive and anesthetic properties, increasingly implicated in recreational use and drug-facilitated crimes. Its effects are amplified when combined with alcohol, posing diagnostic and therapeutic challenges in emergency settings due to its rapid metabolism and frequent co-ingestion with other substances. We describe two cases of severe GHB intoxication in adult male patients resulting in bradycardia, respiratory depression, and decreased level of consciousness requiring orotracheal intubation and intensive care unit (ICU) stabilization. Awareness of GHB’s clinical profile is essential for timely diagnosis and treatment. Given its association with drug-facilitated sexual assault and the potential for life-threatening outcomes, healthcare professionals must maintain a high index of suspicion and initiate appropriate supportive measures promptly.

## Introduction

Gamma-hydroxybutyrate (GHB) is both a precursor and a metabolite of the gamma-aminobutyric acid (GABA) neurotransmitter, with central nervous system (CNS) depressant properties ​​[1]. Initially synthesized as a GABA analog, it began to be used recreationally as a psychoactive stimulant in the early 1990s, a period during which the first reports of theft and sexual assault emerged, particularly involving vulnerable individuals who unknowingly ingested GHB mixed into alcoholic beverages [2]. Its effects are similar to those of ketamine or alcohol. Prior to its recreational use, GHB was introduced into clinical practice as an anesthetic agent in the 1960s [3].

The United Kingdom has one of the highest rates of GHB use, with an annual prevalence of one person every 1,000, a figure also observed in Australia [4]. The drug is consumed both recreationally and in “chemsex” settings, sexual activities prolonged by the use of psychoactive substances [5]. Recreational users tend to be younger (average age of 23) and typically use GHB in party and club environments. In contrast, individuals with GHB dependence are generally older (average age of 30), often socially isolated, and more likely to consume the drug at home [6].

In Brazil, GHB is also known by various street names such as “liquid ecstasy,” “Gisele,” “G-drug,” and “Cinderella’s Goodnight,” owing to its liquid form, which is odorless and tasteless. Along with ketamine and benzodiazepines, it is classified as a crime-facilitating drug due to its sedative and amnestic properties. Many GHB users also consume stimulants such as amphetamines and cocaine. These individuals often use GHB to counteract the stimulating effects of these substances or to manage stimulant-induced insomnia. To date, there are no epidemiological studies on GHB use in Brazil, unlike in other parts of the world [7].

GHB has a rapid onset of action, typically beginning 15-30 minutes after ingestion, with peak serum levels around one hour and effects lasting approximately three hours. Initial effects include euphoria and increased libido, which, depending on the dose, can progress to nausea, vomiting, respiratory depression, and decreased consciousness. These effects are significantly intensified when GHB is combined with alcohol [8]. GHB overdose is relatively rare and is associated with low mortality. There is no known antidote, so the management of acute intoxication relies on supportive care, risk factor control, and preventive airway management [9]. Although uncommon, chronic use can lead to dependence, with users requiring progressively higher doses to achieve the desired effects. Abrupt discontinuation may trigger a withdrawal syndrome characterized by tremors, agitation, and delirium [9].

We present a case series about two patients who ingested high doses of GHB, leading to hemodynamic instability and acute kidney failure, requiring dialysis and intensive care unit (ICU) monitoring.

## Case presentation

A woman called emergency medical services after finding two young adults with abnormal behavior in a public park. Upon arrival, one patient was found to have a decreased level of consciousness (Glasgow Coma Scale score of 3), while the other was still awake. After initial stabilization, both patients were taken to the emergency department for urgent care.

**Figure 1:**
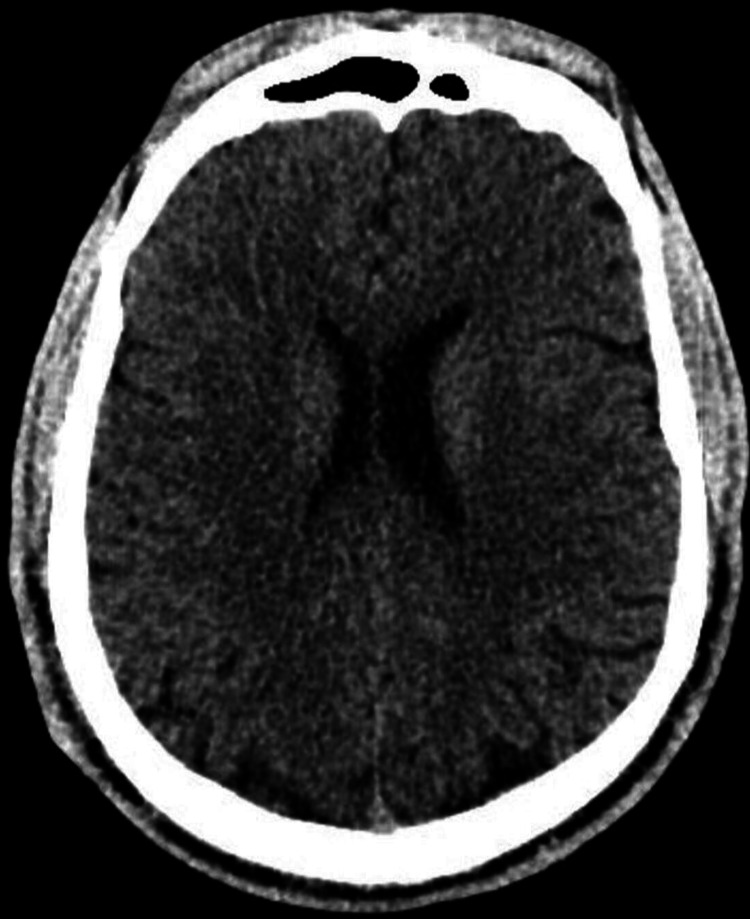
Head CT scan of the first patient (FR) This non-contrast axial head CT shows a normal study at the level of the frontal horns of the lateral ventricles. The frontal sinuses are well-aerated anteriorly, and the cerebral hemispheres appear symmetric with preserved gray-white matter differentiation, indicating no evidence of acute ischemia or cerebral edema. The lateral ventricles are normal in size and shape, with no signs of hydrocephalus or midline shift. The septum pellucidum is midline and intact. There is no evidence of intracranial hemorrhage, mass effect, or extra-axial collections. CT: computed tomography

**Figure 2:**
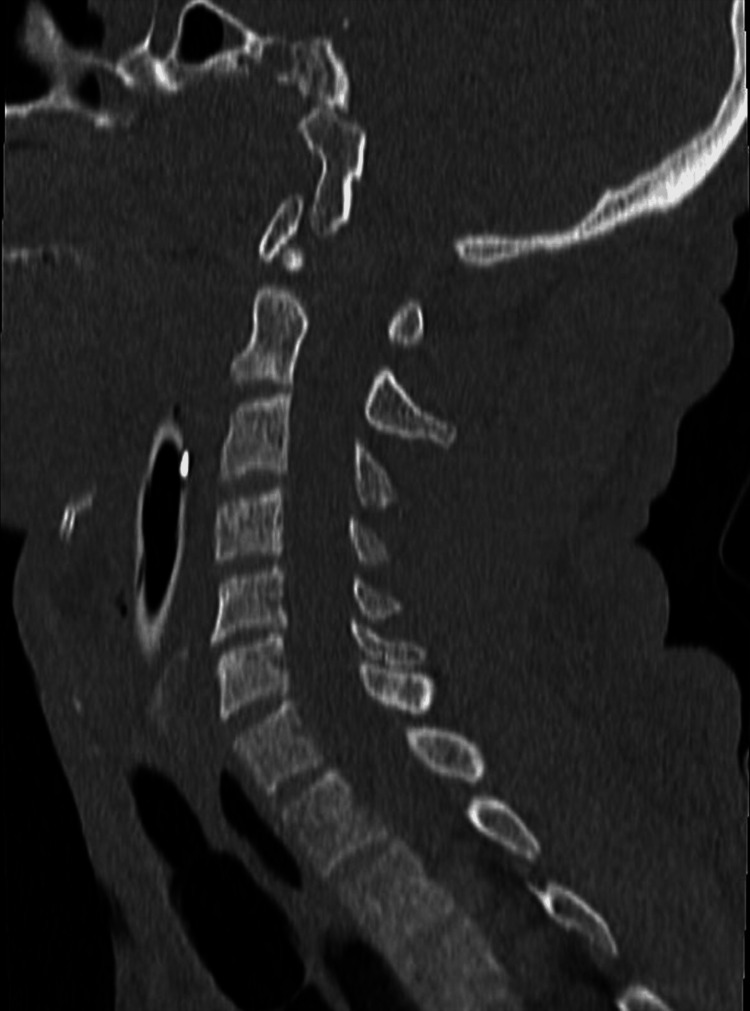
Neck CT scan of the first patient (FR) This sagittal CT image of the cervical spine demonstrates a normal osseous anatomy. The vertebral bodies are well-aligned, with preserved vertebral height and intact cortical margins, showing no signs of compression fractures or lytic lesions. The intervertebral disc spaces are maintained throughout, suggesting the absence of significant degenerative disc disease. The spinous processes, laminae, and pedicles appear intact and symmetric. CT: computed tomography

**Figure 3:**
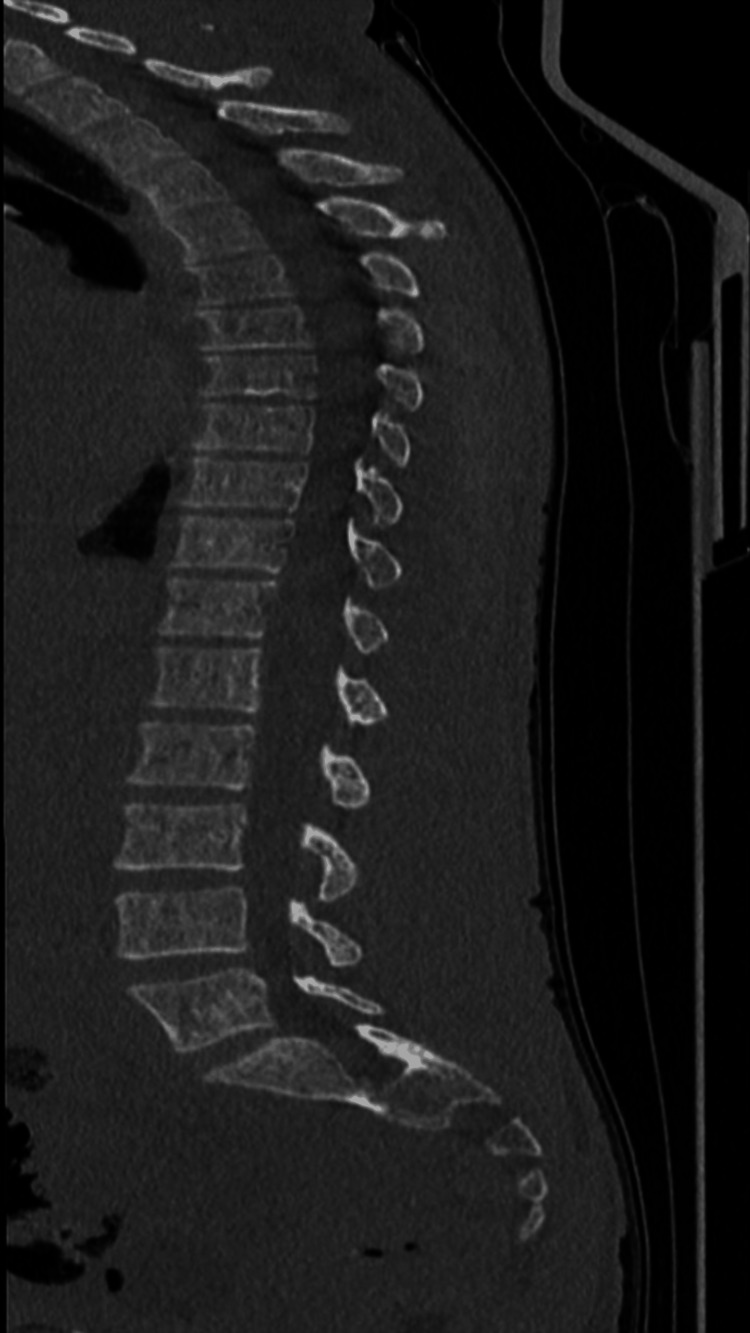
Spine CT scan of the first patient (FR) This sagittal CT image of the spine demonstrates a normal osseous anatomy from the thoracic spine through the sacrum. The vertebral bodies are well-aligned, with preserved vertebral height and intact cortical margins, showing no signs of compression fractures or lytic lesions. The intervertebral disc spaces are maintained throughout, suggesting the absence of significant degenerative disc disease. CT: computed tomography

The first patient (FR), a 25-year-old man, presented with bradycardia (heart rate: 44 beats per minute (BPM)), hypotension (64/43 mmHg), bradypnea (respiratory rate: 10 breaths per minute), desaturation (82% in room air), and altered level of consciousness upon initial assessment, being intubated and receiving two ampoules of atropine and 2 L of saline solution. Upon arrival at the hospital, he remained intubated, and norepinephrine was initiated, which normalized his vital signs within 20 minutes. His pupils were mid-sized, fixed, and non-reactive. The main diagnostic hypothesis was trauma or intoxication by undetermined psychoactive substances. Laboratory results yielded a mild metabolic acidosis (Table 1). The patient arrived under the protocol for a patient found with altered level of consciousness in a public area, requiring imaging examinations, including computed tomography (CT) scan of the head and cervical, thoracic, and lumbar spine, as well as an electroencephalogram, which showed no significant findings (Figures 1-3).

**Table 1:** Initial laboratory investigations of the first patient (FR) Hb: hemoglobin, Ht: hematocrit, pCO_2_: partial pressure of carbon dioxide, HCO_3_⁻: bicarbonate, BE: base excess, INR: international normalized ratio, Cr: creatinine, Ur: urea, Na: sodium, K: potassium

Test	Patient value	Reference range
Hb	16.8 g/dL	13-18 g/dL
Ht	46.8%	40%-52%
Leukocyte	7,610×10^3^/µL	3,800-11,000×10^3^/µL
Platelet count	204,000×10^3^/µL	140,000-450,000×10^3^/µL
pH (blood)	7.3	7.35-7.45
pCO_2_	39 mmHg	32-45 mmHg
HCO_3_⁻	19.8 mmol/L	20-26 mmol/L
BE	-5.6 mmol/L	-3 to +3 mmol/L
Lactate	1.8 mmol/L	0.5-2.2 mmol/L
INR	0.77	1.0-1.3
Cr	0.83 mg/dL	0.7-1.3 mg/dL
Ur	21 mg/dL	10-50 mg/dL
Na	134 mEq/L	136-145 mEq/L
K	4.0 mEq/L	3.5-5.1 mEq/L

The patient had a history of bipolar disorder and was using lithium, sertraline, and clonazepam. Family members reported regular cocaine use. During hospitalization, he developed refractory metabolic acidosis (pH: 7.1, HCO_3_⁻: 10, and anion gap: 10) and polyuria (6 L in the last four hours) with suspected lithium intoxication and underwent hemodialysis using FX80 and FX100 capillaries six hours after being found. On the same day, a few hours after dialysis, he was extubated and could maintain normal vital signs.

The other patient (LB), a 43-year-old man, was initially awake, with rapid and loud speech. After 10 minutes, he developed bradycardia (heart rate: 50 BPM), hypotension (70/58 mmHg), bradypnea (RR: 9 breaths per minute), and desaturation (88% in room air). After initial stabilization with 1 L of normal saline infusion and intubation, the patient developed recurrent seizures during transport by ambulance, receiving 10 mg of intramuscular midazolam. The patient had a past medical history of HIV (using regular medication) and testosterone use for athletic purposes. The main diagnostic hypothesis was intoxication by undetermined psychoactive substances. Initial laboratory evaluation indicated a respiratory and metabolic acidosis (Table 2).

**Table 2:** Initial laboratory investigations of the second patient (LB) Hb: hemoglobin, Ht: hematocrit, pCO_2_: partial pressure of carbon dioxide, HCO_3_⁻: bicarbonate, BE: base excess, INR: international normalized ratio, Cr: creatinine, Ur: urea, Na: sodium, K: potassium

Test	Patient value	Reference range
Hb	14.2 g/dL	13-18 g/dL
Ht	40%	40%-52%
Leukocyte	12,590×10^3^/µL	3,800-11,000×10^3^/µL
Platelet count	305,000×10^3^/µL	140,000-450,000×10^3^/µL
pH (blood)	7.15	7.35-7.45
pCO_2_	50.1 mmHg	32-45 mmHg
HCO_3_	17.8 mmol/L	20-26 mmol/L
BE	-10.3 mmol/L	-3 to +3 mmol/L
Lactate	0.85 mmol/L	0.5-2.2 mmol/L
INR	0.96	1.0-1.3
Cr	0.88 mg/dL	0.7-1.3 mg/dL
Ur	18.5 mg/dL	10-50 mg/dL
Na	135 mEq/L	136-145 mEq/L
K	4.2 mEq/L	3.5-5.1 mEq/L

**Figure 4:**
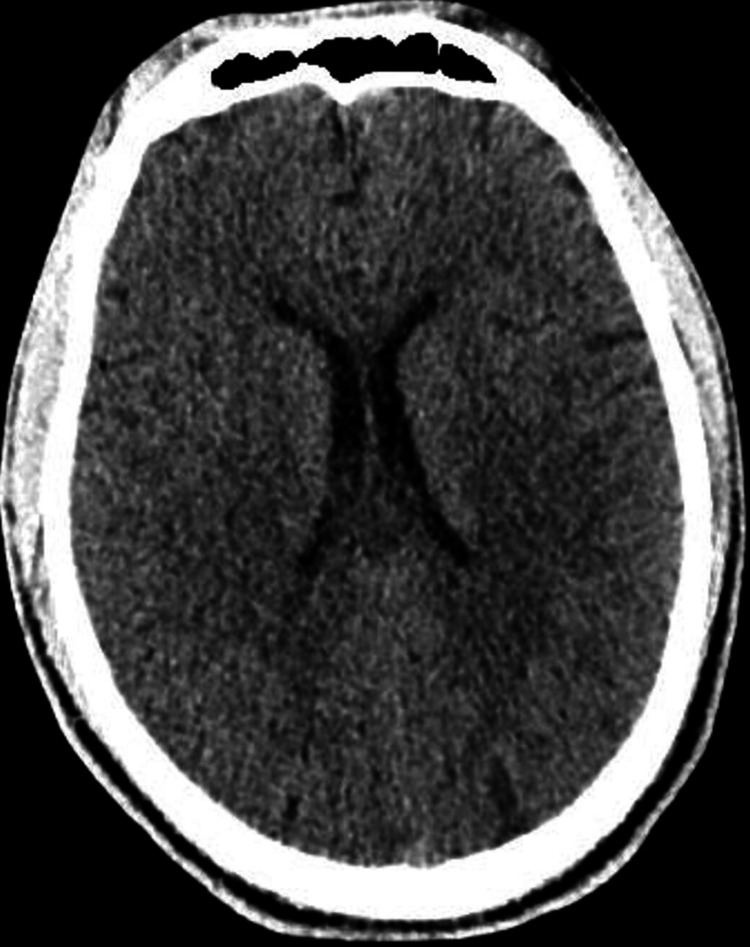
Head CT scan of the second patient (LB) This non-contrast axial head CT shows a normal study at the level of the frontal horns of the lateral ventricles. The frontal sinuses are well-aerated anteriorly, and the cerebral hemispheres appear symmetric with preserved gray-white matter differentiation, indicating no evidence of acute ischemia or cerebral edema. The lateral ventricles are normal in size and shape, with no signs of hydrocephalus or midline shift. The septum pellucidum is midline and intact. There is no evidence of intracranial hemorrhage, mass effect, or extra-axial collections. CT: computed tomography

**Figure 5:**
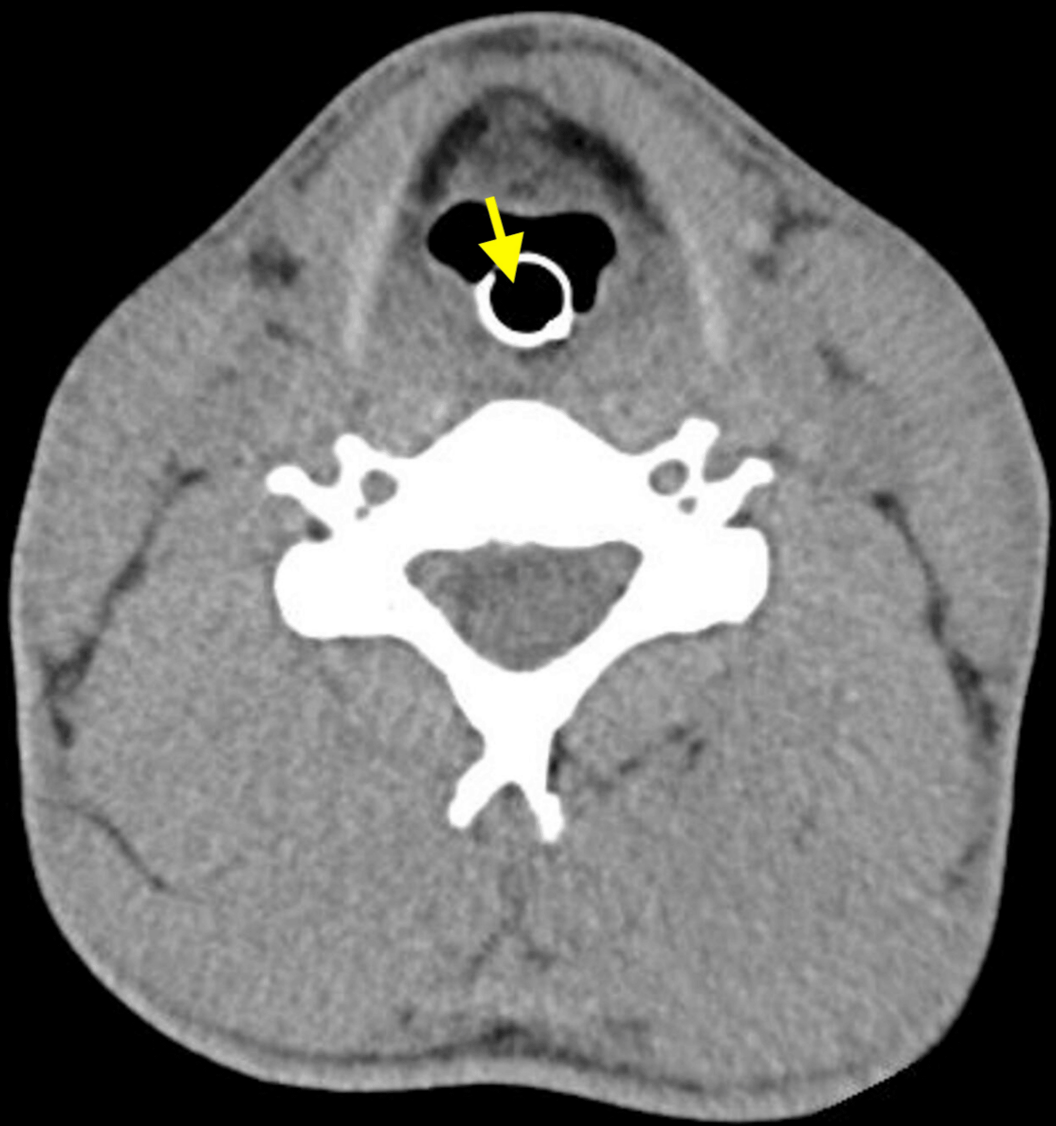
Neck CT scan of the second patient (LB) Axial image of a neck CT scan without contrast, localized at the C5 level. No abnormalities are found, rather for the presence of an orotracheal tube inside the trachea (yellow arrow). The cervical vertebra is preserved, without signs of fracture or trauma. There are no signs of neck hematoma. CT: computed tomography

**Figure 6:**
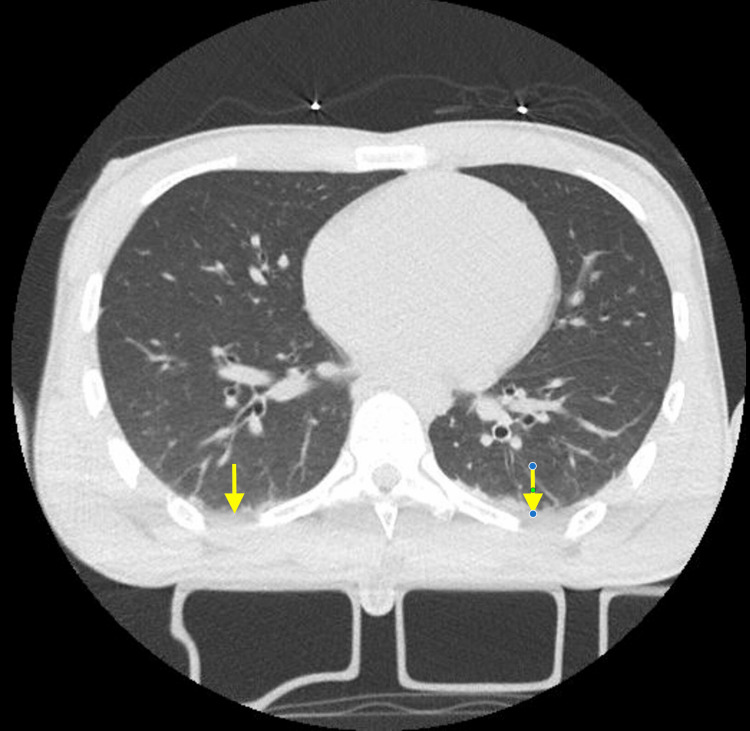
Chest CT scan of the second patient (LB) This axial chest CT image demonstrates a lung window view at the level of the lower thorax, showing mild bilateral base pleural effusion and atelectasis (yellow arrows). There is also a normal pulmonary parenchyma without evidence of consolidation, ground-glass opacities, or masses. The cardiac area is fully visualized without signs of cardiomegaly. CT: computed tomography

A head CT scan showed no acute changes, and a cervical CT scan showed no abnormalities, while a chest CT scan revealed mild bilateral pleural effusion and atelectasis (Figures 4-6). LB received treatment for status epilepticus with phenytoin, as well as ventilatory and hemodynamic support, with the administration of norepinephrine, vasopressin, and hydrocortisone. Due to suspected aspiration pneumonia, an antibiotic regimen with ceftriaxone, clindamycin, and azithromycin was initiated. After stabilization, he was transferred to the intensive care unit (ICU), and one day later, he was extubated.

Following a psychiatric consultation, both patients reported use of approximately 10 mL of GHB minutes before being found unconscious. It was their first experience using this substance after they bought it illegally from a new drug dealer. They showed clinical improvement and a desire to achieve abstinence. The two patients were discharged three days apart, with outpatient follow-up appointments scheduled for them. LB was discharged on chlorpromazine 100 mg and diazepam 5 mg at night, while FR was sent home with risperidone 0.5 mg and valproic acid 500 mg at night.

## Discussion

In this case report, we presented two patients who unintentionally overdosed on GHB. The reported symptoms are similar to those described in the literature and other case reports around the world [10,11]. Although recreational use of GHB is rarer than with other substances, it is important for healthcare providers to remember its main symptoms and keep it as a differential diagnosis, especially in emergency settings, since it is not detected by regular urine drug screenings and has the potential to lead to death if not treated.

The main symptoms of GHB intoxication are nonspecific and can vary depending on individual tolerance, dose, and route of administration. Relaxation, ataxia, disorientation, euphoria, confusion, and hallucinations are the most commonly reported signs of GHB intoxication. However, emergency department physicians tend to underestimate and underrecognize GHB abuse based on clinical observations alone [12]. Additionally, concomitant alcohol intoxication is common, making diagnosis more challenging, especially in cases of unintentional intoxication, since alcohol can worsen symptoms and increase the risk of GHB overdose [13]. At low doses (<10 mg/kg), presentation may include mild clinical effects similar to light alcohol intoxication, such as euphoria and short-term anterograde amnesia. At 20-30 mg/kg, the presentation may include excessive drowsiness or myoclonus. At a dose of 50 mg/kg, the patient may present as comatose. An overdose is considered at doses >50 mg/kg, and the patient may present in a coma with bradycardia and/or respiratory depression [14].

When GHB intoxication is suspected, it is important to inquire about the dosage used, route of administration (usually oral, but it can be injected intravenously), concomitant use of alcohol or other drugs, use of antiretroviral medications for HIV (as they can alter its metabolism and bioavailability), and recent sexual history, including sexual assault. As for the GHB dosage, it can be difficult to quantify, since patients may report a “full cap” or milliliters of a mixed solution in which concentrations vary depending on the supplier and the batch, and involuntarily intoxicated patients will not know the dosage at the time of ingestion. The diagnosis is based on clinical presentation and can be confirmed by laboratory tests. GHB has a short half-life; therefore, blood and urine samples should be collected as soon as possible, especially in cases of sexual assault. Serum concentrations of GHB can range from 80 to 200 mg/L after recreational use, and levels ≥ 300 mg/L may lead to cardiorespiratory depression, shock, and even death. Urine tests may have a detection window of 3-4 hours, which is therefore longer than that of blood tests [15].

Treatment for mild GHB intoxication consists of clinical support, including respiratory and cardiovascular support, monitoring of vital signs, and an electrocardiogram. Airway protection with orotracheal intubation may be necessary in cases of decreased consciousness. There are no medications available to reverse GHB intoxication or sedation. Decontamination with gastric lavage or activated charcoal is generally not beneficial due to GHB’s rapid absorption; however, it may be considered in alert patients for detoxification of other associated substances. Central nervous system (CNS) depression usually resolves within 1-3 hours, but the patient may become agitated and require restraint after regaining consciousness. Full recovery typically occurs within 4-8 hours, but hemodialysis might be required, especially in cases of metabolic acidosis [14].

In this case of GHB intoxication, one patient presented with severe, persistent metabolic acidosis that did not respond to standard bicarbonate therapy. Hemodiafiltration was initiated due to the refractory nature of the acidemia, despite the ingestion being initially unconfirmed, and due to the suspicion of lithium toxicity. GHB is a low-molecular-weight compound with minimal protein binding and a small volume of distribution, making it amenable to removal via extracorporeal techniques. The use of hemodiafiltration is described in one previous case report in the literature [16]. The present case is another one that highlights the utility of hemodiafiltration in managing profound acidemia resulting from massive GHB ingestion.

## Conclusions

GHB intoxication presents a unique challenge in clinical practice due to its nonspecific symptoms, rapid metabolism, and frequent co-ingestion with alcohol, which can exacerbate its sedative effects. Although its recreational use is less common compared to other substances, the potential for severe outcomes, including death, warrants heightened awareness among healthcare professionals, particularly in emergency settings where timely diagnosis and supportive care are critical. Additionally, the substance’s association with sexual assault highlights the importance of prompt toxicological testing and careful medical interview, especially in vulnerable populations.
